# Characteristic fast H^−^ ion conduction in oxygen-substituted lanthanum hydride

**DOI:** 10.1038/s41467-019-10492-7

**Published:** 2019-06-12

**Authors:** Keiga Fukui, Soshi Iimura, Tomofumi Tada, Satoru Fujitsu, Masato Sasase, Hiromu Tamatsukuri, Takashi Honda, Kazutaka Ikeda, Toshiya Otomo, Hideo Hosono

**Affiliations:** 1 Laboratory for Materials and Structures, Tokyo Institute of Technology, Yokohama, 226-8503 Japan; 2 Materials Research Center for Element Strategy, Tokyo Institute of Technology, Yokohama, 226-8503 Japan; 3 Institute of Materials Structure Science, High Energy Accelerator Research Organization (KEK), Tsukuba, 305-0801 Japan; 4 Department of Materials Structure Science, The Graduate University for Advanced Studies, Tsukuba, 305-0801 Japan

**Keywords:** Solid-state chemistry, Batteries, Electrochemistry

## Abstract

Fast ionic conductors have considerable potential to enable technological development for energy storage and conversion. Hydride (H^−^) ions are a unique species because of their natural abundance, light mass, and large polarizability. Herein, we investigate characteristic H^−^ conduction, i.e., fast ionic conduction controlled by a pre-exponential factor. Oxygen-doped LaH_3_ (LaH_3__−2*x*_O_*x*_) has an optimum ionic conductivity of 2.6 × 10^−2^ S cm^−1^, which to the best of our knowledge is the highest H^−^ conductivity reported to date at intermediate temperatures. With increasing oxygen content, the relatively high activation energy remains unchanged, whereas the pre-exponential factor decreases dramatically. This extraordinarily large pre-exponential factor is explained by introducing temperature-dependent enthalpy, derived from H^−^ trapped by lanthanum ions bonded to oxygen ions. Consequently, light mass and large polarizability of H^−^, and the framework comprising densely packed H^−^ in LaH_3__−__2*x*_O_*x*_ are crucial factors that impose significant temperature dependence on the potential energy and implement characteristic fast H^−^ conduction.

## Abstract (web summary)

Hydride ions are promising for energy storage since they are abundant, lightweight, and highly mobile, but ionic conductivity should be improved. Here the authors achieve fast hydride ion conductivity in a mixed-anion compound by tuning oxygen content.

## Introduction

Hydride (H^−^) ions are electrochemically attractive charge carriers not only because they have small ionic radii and a valence electron shell suitable to ionic conduction in solids but also because of the high standard redox potential of H_2_/H^−^ (−2.3 V). These characteristics make H^−^ ions promising for applications in next-generation electrochemical energy storage systems with high voltage and high energy density. To date, H^−^ conduction has been reported in solid hydrides and oxyhydrides^1–3^. However, high H^−^ conduction has been achieved only at relatively high temperatures (0.04–0.2 S cm^−1^ at 450 °C–630 °C for the high-temperature phase of BaH_2_)^2^. Unlocking a wider range of applications in energy storage systems requires materials with a high H^−^ conductivity at lower temperatures.

Hydrogen is the lightest element. Over the years, diffusion of hydrogen in metals and that of protons (H^+^) in hydrogen-bonded liquids have attracted significant research attention both experimentally and theoretically. The diffusion coefficient and conductivity have many peculiar features in terms of magnitude and their dependence on isotope mass and temperature. These features are now understood as a consequence of quantum-mechanical processes originating from small mass^4^.

In addition to the smallest mass, a unique and important feature of H^−^ is its large electronic polarizability^5^. H^−^ comprises one proton and two electrons. Contrary to a single proton with no electrons, H^−^ is regarded as a soft anion due to its large electronic polarizability, which arises from its considerably small electron affinity. O^2−^ and F^−^ have ionic radii of 1.2–1.3 Å, similar to that of H^6^. However, the large polarizability of H^−^ is in sharp contrast with those of anions, which have low polarizability and are typically considered as hard anions^7^. In terms of ionic conduction, the polarizability of counterpart ions or the framework is widely recognized as a key factor that reduces the activation energy of ion hopping^8^. For example, Li^+^ conduction in sulfides is much faster than that in oxides^9^. Similarly, high O^2−^ and F^−^ conduction occurs in solids based on highly polarizable cations, e.g., δ-Bi_2_O_3_ and PbF_2_, respectively, rather than in isostructural Y_2_O_3_-stabilized ZrO_2_ and CaF_2_^10^. However, the polarizability of mobile ions and the concomitant effects on ionic conduction have yet to be elucidated.

**Fig. 1:**
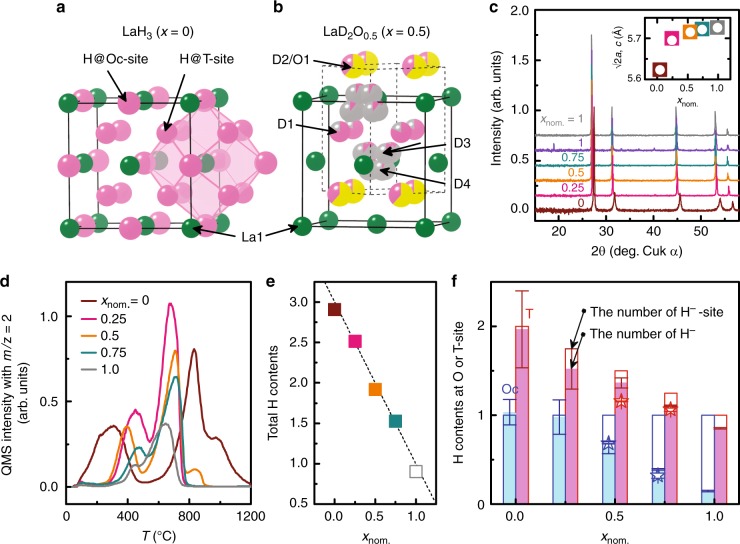
Structural and compositional analyses for LaH_3-2*x*_O_*x*_ and LaD_3-2*x*_O_*x*_. **a** Crystal structure of LaH_3_. **b** Crystal structure of LaD_2_O_0.5_ determined by NPD. Solid and dashed lines represent the unit cells of the fcc and tetragonal *P*4/*nmm* structures, respectively. Green, pink, yellow, and gray colors denote the fractions of La^3+^, D^−^, O^2−^, and vacancies at each crystallographic site, respectively^38^. **c** *x*_nom_ dependence of XRD patterns measured after the conductivity measurement heated up to 340 °C. At *x*_nom_ = 1.0, the profile shown as gray (before conductivity measurement) changes to purple (after conductivity measurement). The inset shows the lattice parameters *a* multiplied by √2 and *c* as functions of *x*_nom_, as obtained from Rietveld refinement using the *P*4/*nmm* structure. The open circle and filled square show √2*a* and *c*, respectively. **d** Thermal-desorption profiles of *m*/*z* = 2 (H_2_^+^). These profiles are taken from samples after the electrical conductivity measurements, except for *x*_nom_ = 1, for which the data are collected before the electrical conductivity measurement. **e** Analyzed hydrogen content as a function of *x*_nom_. All values are calculated from the TDS profiles shown in panel **d**. Dashed line is *y* = 3−2*x*_nom_. **f** Hydrogen content at Oc (blue) and T sites (red) as a function of *x*_nom_. Open star symbols at *x*_nom_. = 0.5 and 0.75 show deuterium content as obtained by NPD. Open blue and red bars show the calculated available number of Oc and T sites for hydrogen, respectively, assuming that oxygen occupies only T sites. Solid blue and red bars show the number of hydrogen atoms at Oc and T sites measured by TDS, respectively. The error bars show the standard deviation calculated from peak fitting

In this paper, we reveal a characteristic fast H^−^ conduction arising from its light mass and high polarizability, i.e., fast ionic conduction controlled by a pre-exponential factor. To demonstrate this, we choose the material system LaH_3_, in which H^−^, rather than La^3+^, is the nearest neighbor of each H^−^. As shown in Fig. 1, LaH_3_ crystalizes in a face-centered-cubic (fcc) structure composed of a face-sharing La_4_ tetrahedron and a La_6_ octahedron. Two of the three H^−^ per La occupy the tetrahedral site (T site) and the other is located at the octahedral site (Oc site). The minimum separation between H^−^ ions at the Tet and at the Oc sites is only 2.4 Å, and the eight H^−^ ions at the T sites and six at the Oc sites form of a rhombic dodecahedron encapsulating a La^3+^ (Fig. 1a).

Partial substitution of O^2−^ in the H^−^ site of LaH_3_ (LaH_3−2*x*_O_*x*_) is required to create a vacancy at either the T or Oc sites and to suppress electronic conduction by deepening the donor level (relative to the vacuum level), which is generated by a tiny deficiency in hydrogen content. Because the ionic radius of H^−^ is similar to that of O^2−^, both ions have a wide range of solubility in various compounds such as LaFeAsO and BaTiO_3_, where the substituted H^−^ is stabilized at the center of a positively charged polyhedron^11,12^.

## Results

### Crystal structure and chemical composition

Figure 1b shows the crystal structure of LaD_2_O_0.5_ [the nominal *x* (*x*_nom_) is 0.5], as determined by a room-temperature neutron powder diffraction (NPD) measurement. Although the space group is tetragonal *P*4/*nmm*, which is a subgroup of fcc, the tetragonality is quite small (*c*/√2*a* = 0.9991, see Supplementary Fig. 1 and Supplementary Table 1 for detailed information on the structure)^13^. We also applied a Rietveld analysis by using another structure model with a disordered oxygen at the T site of the fcc lattice; however, the goodness of fit was worse than that for the tetragonal model. Moreover, electron diffraction done with a transmission electron microscope detected no additional spots derived from the fcc superstructure (see Supplementary Fig. 2), suggesting that oxygen atoms are ordered over a very long range (We conducted the neutron pair distribution function measurement on LaD_3__−__2*x*_O_*x*_ with *x*_nom_ = 0.25 at 300 K to check the local structure. The preliminary result of fitting using the *P*4/*nmm* model to the data sufficiently reproduces the neutron pair distribution function pattern.). The LaD_2_O_0.5_ structure contains four symmetrically different deuterium positions: D1 and D2 correspond to the T site of LaH_3_ (the T_D1_ and T_D2_ sites, respectively), whereas D3 and D4 correspond to the Oc site (the Oc_D3_ and Oc_D4_ sites, respectively). Contrary to the deuterium distributed to both the T and Oc sites, the oxygen dopant preferentially occupies the T_D2_ site. As reported for undoped LaH_3_, the Oc_D3_ and Oc_D4_ sites are not at the center but are displaced by 0.85 and 0.57 Å from the center of the La_6_ octahedron toward the T_D1_ and T_D2_ sites, respectively. The unit cell of the *P*4/*nmm* lattice contains two metal sites, four T sites, and two Oc sites for anions. According to the results of Rietveld refinement for LaD_2.0_O_0.5_ and LaD_1.5_O_0.75_, the chemical-composition ratios in the unit cell of the *P*4/*nmm* are La: D: O = 2 (fixed): 3.67(1): 1.32(4) for LaD_2.0_O_0.5_ and La: D: O = 2 (fixed): 2.76(4): 1.61(1) for LaD_1.5_O_0.75_ which are close to the nominal ratios each other.

Figure 1c shows X-ray diffraction (XRD) patterns for several values of the *x*_nom._. These data were collected after conductivity measurements at temperatures up to 340 °C. All XRD patterns except for *x*_nom_ = 1.0 remain unchanged upon heating to high temperatures and can all be indexed to the *P*4/*nmm* structure. At *x*_nom_ = 1.0, the as-prepared sample synthesized at 750 MPa forms a fcc structure but changes after the conductivity measurement to other tetragonal structure which is reported to be an ambient-pressure phase^14,15^. Rietveld refinements from the XRD profiles indicate that the oxygen preferentially occupies the T site, which is consistent with the NPD result. The refined lattice parameter *a* multiplied by √2 and *c* are plotted in the inset of Fig. 1c. Both values are close each other, indicating the small tetragonality. As *x*_nom._ increases, the two values gradually increases because of oxygen incorporation at the T site with the larger ionic radius of O^2−^ than that of H^−^ in LaH_3−2*x*_O_*x*_^6,16^.

We use thermal-desorption spectroscopy (TDS) to examine the dopability and occupation of H^−^ in each site. Figure 1d shows TDS profiles of LaH_3−2*x*_O_*x*_. Stoichiometric LaH_3_ has two distinct desorption peaks: one near 300 °C and another near 800 °C. The area ratio of the low- to high-temperature peaks is close to 1:2, indicating that these peaks are assigned to the desorption of hydrogen occupying the Oc and the T sites, respectively. The total amount of hydrogen desorbed from each sample is estimated by integrating the area of the two peaks (Fig. 1e), and the result is consistent with the fractional hydrogen content 3−2*x*_nom_. Hereinafter we denote by *x* the oxygen content determined by this analysis.

For each *x*_nom_, hydrogen content at the Oc and at the T sites is calculated from the areas of the low- and high-temperature peaks, respectively, from the TDS profile (see Fig. 1f). In this figure, hydrogen content at the O and T sites is compared with the number of O and T sites available for hydrogen, which is estimated by considering the fact that oxygen occupies only the T site. Thus, the number of T sites decreases with oxygen content (*x*_nom_), whereas the number of Oc sites remains unchanged. Oxygen substituted for H^−^ at a T site behaves as an O^2−^ ion. To maintain charge neutrality, a hydrogen vacancy is generated at a T or Oc site. Figure 1f suggests that the vacancy forms mainly at the Oc site. At *x*_nom_ = 0.5, the deuterium content at the Oc and the T sites estimated by NPD (denoted by open stars) is compared with the hydrogen content from TDS; the results are consistent.

### Conductivity

**Fig. 2:**
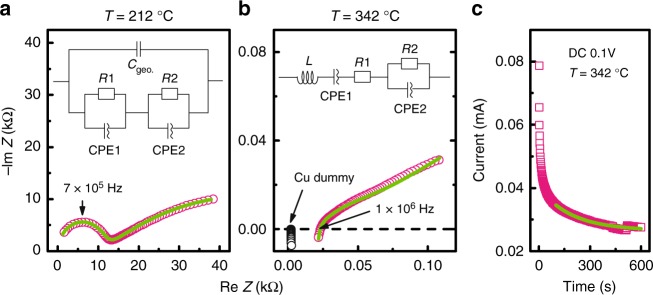
Alternating current impedance spectra and direct current polarization data. **a**, **b** Complex impedance plots of sample with *x* = 0.24 at *T* = 212 °C and *T* = 342 °C. The corresponding equivalent circuits used for the simulation are shown in the insets. *C*_geo_, *R*, CPE, and *L* are parasitic capacitance, resistance, constant phase element, and inductance, respectively. Open black circles show the result for Cu. **c** Current as a function of time upon application of 0.1 V constant voltage at *x* = 0.25 and *T* = 342 °C

Figure 2a shows the results of AC impedance spectroscopy for a sample with *x* = 0.24 at *T* = 212 °C. A typical half-circle response appears in the higher-frequency region, which corresponds to the bulk resistance. The curve in the low-frequency region derives from ionic motion at grain boundaries and/or interfaces between sample and electrode. The curve fits are also plotted in the figure. We use an equivalent circuit (see inset of Fig. 2a) with a parasitic capacitance *C*_geo_ to reflect the parallel configuration of electrodes, which reproduces well the electric responses. As the temperature increases to 342 °C, the half-circle response fades because the sample resistance becomes comparable to that of the inductance component (Fig. 2b). However, the spectrum differs totally from those of metallic Cu, which is shown as black circles, and is successfully simulated by the equivalent circuit that includes a wire inductance *L*. Therefore, the conductivity estimated from the resistance *R*1 correctly traces the bulk conductivity. To examine the effect of grain boundary on the conductivity, we prepared a denser sample by using a different sample cell for the high-pressure synthesis, and compared the conductivity and grain boundary with those of sample synthesized at 750 MPa. The schematic of sample cell we used, images of the grain boundary and result of conductivity are shown in Supplementary Figs. 3–6 and Supplementary Table 2. The ionic conductivity *σ*_i_ at *T* = 212 °C and 342 °C is calculated to be 4.1 × 10^−5^ and 2.6 × 10^−2 ^S cm^−1^, respectively. For hydride-ion conductors, the latter value is the highest reported to date for intermediate temperatures. Figure 2c displays the results of DC polarization measurements made at *T* = 342 °C. The electronic conductivity *σ*_e_ = 4.85 × 10^−5^ S cm^−1^ estimated from the steady-state current is two orders of magnitude less than *σ*_i_, so the main contribution to the observed conductivity is ionic [transport number *t*_i_ = *σ*_i_ / (*σ*_i_ + *σ*_e_) = 0.998].

**Fig. 3:**
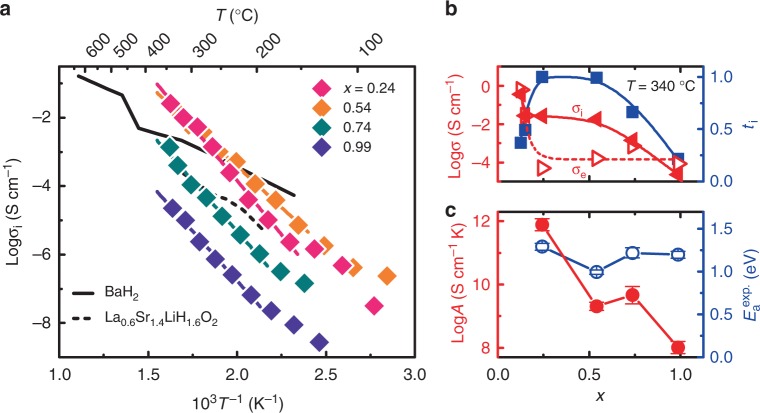
*x* dependence of ionic and electronic conductivities. **a** Arrhenius plots of LaH_3-2*x*_O_*x*_ and H^−^ conductors reported to date^2, 3^. **b** Conductivity (red) and ion transport number *t*_i_ (blue) as functions of oxygen content *x* at *T* = 340 °C. Red open and solid triangles are electronic (*σ*_e_) and ionic (*σ*_i_) conductivities, respectively. **c** Logarithm of pre-exponential factor *A* (red) and activation energy *E*_a_^exp^ (blue) as functions of oxygen content *x*, as derived from Eq. 1

Figure 3a shows the ionic conductivity of LaH_3-2*x*_O_*x*_ (0.24 ≤ *x* ≤ 0.99) and other H^−^ conductors (BaH_2_ and La_1.6_Sr_0.4_LiH_0.6_O_3_)^2,3^. The optimum ionic conductivity of 2.6 × 10^−2^ S cm^−1^ occurs at *x* = 0.24 and *T* = 342 °C. Figure 3b shows the quantities *σ*_i_, *σ*_e_, and *t*_i_ as a function of oxygen content *x* at *T* = 340 °C. Both *σ*_i_ and *σ*_e_ decrease as *x* increases, whereas *t*_i_ attains a maximum > 0.99 for 0.24 < *x* < 0.54. For the lightly doped region (i.e., *x* < 0.24), the small *t*_i_ is ascribed to the large *σ*_e_, whereas the drop in *t*_i_ for *x* > 0.54 is attributed to the small *σ*_i_. The ionic conductivity *σ*_i_ of the solid usually obeys the Arrhenius equation:

1 $$\sigma _{\mathrm{i}}T{\mathrm{ = }}A{\mathrm{exp}}\left( {{\mathrm{ - }}E_{\mathrm{a}}^{{\mathrm{exp}}}{\mathrm{/}}k_{\mathrm{B}}T} \right){\mathrm{,}}$$

where *E*_a_^exp^, *A*, and *k*_B_ are the activation energy, pre-exponential factor, and Boltzmann constant, respectively. The activation energy *E*_a_^exp^ and pre-exponential factor *A* estimated by using Eq. (1) are plotted as functions of oxygen content *x* in Fig. 3c. The activation energy *E*_a_^exp^ at *x* = 0.24 is estimated to 1.3 eV, which is almost double that of reported H^−^ conductors, and *A* = 10^12^ S cm^−1^ K is several orders of magnitude larger than what has heretofore been reported^2,3^. Upon increasing *x*, *E*_a_^exp^ remains unchanged, whereas *A* decreases by four orders of magnitude, which indicates that the pre-exponential factor *A* is the primary factor that determines the high *σ*_i_ and the *x* dependence.

## Discussion

**Fig. 4:**
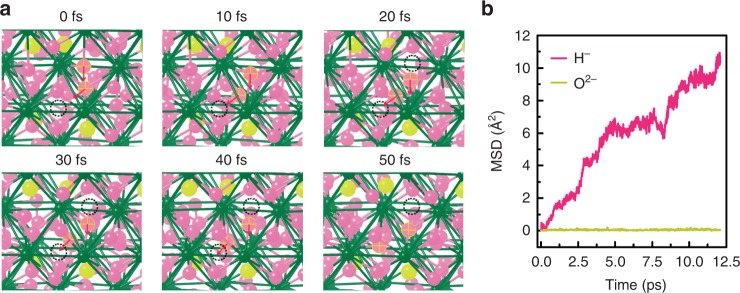
Results of first-principles molecular dynamics simulation. **a** Snapshot of simultaneous two-H^−^ hopping. Green, yellow, and pink spheres represent La, O, and H atoms, respectively. Black circles are vacancy sites. **b** Calculated mean square displacement of H^−^ (pink) and O^2−^ (yellow)

We now discuss the origin of the high activation energy, extraordinarily large pre-exponential factor, and resultant fast ionic conduction in LaH_3−2*x*_O_*x*_. To investigate the ionic conduction from the atomistic point of view, we use a first-principles molecular dynamics (MD) simulation implemented in the Vienna Ab initio Simulation Package (VASP) density-functional code. The MD simulation of LaH_3−2*x*_O_*x*_ at *x* = 0.25 is executed at 400 °C for 12.5 ps. H^−^-ion-hopping events between T and Oc sites occur frequently in the MD simulation (Fig. 4a), whereas O^2−^ ions are stuck to their initial sites. The situation is confirmed by the calculated mean square displacement (MSD) of H^−^ and O^2−^ ions, which is shown in Fig. 4b and indicates that the H^−^ migration is a major component of experimentally observed ionic conduction (We performed a quasi-elastic neutron scattering measurement on LaH_2_O_0.5_ (*x*_nom_ = 0.5), and the preliminary results confirmed a clear quasi-elastic component at 607 K, indicating that the dynamics of hydride ion are present at the temperature.). Allowing the MSD of H^−^ to range from 5 to 12.5 ps and using Einstein’s relationship, we obtain a H^−^-ion conductivity of 1.38 S cm^−1^, which is roughly on line with the measured conductivity of LaH_3-2*x*_O_*x*_ at *x* = 0.24 (2.6 × 10^−2^, 1.4 × 10^−3^, and 2.3 × 10^−5^ S cm^−1^ for 341 °C, 264 °C, and 162 °C, respectively).

Using the structures extracted from the MD simulations, we optimize each structure. Nudged elastic band calculations reveal that the activation energy for H^−^ hopping from Oc sites to T_D1_ sites (which are located far from oxygen; see Fig. 1b) and vice versa range from 30 to 160 meV. The later value is comparable with an activation energy of a long-range diffusion of H^−^ determined by quasi-elastic neutron scattering from LaH_3_ (150 ± 10 meV)^17^, although it is very small compared with the experimentally determined value of 1.3 eV.

**Fig. 5:**
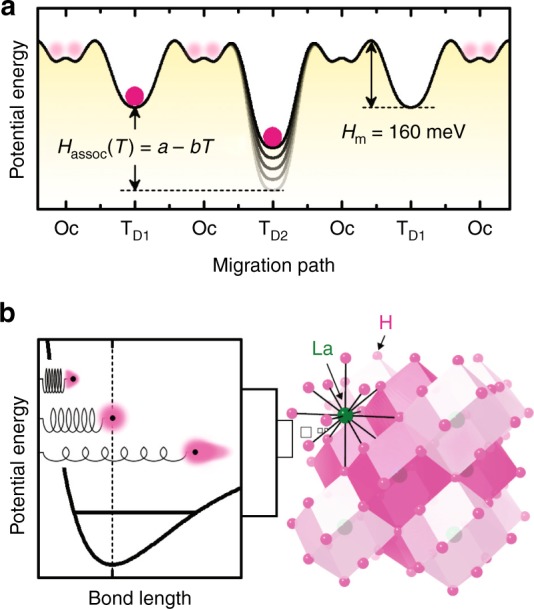
Anharmonicity of H^−^ vibration in LaH_3−2*x*_O_*x*_. **a** Schematic illustration of energy landscape of H^−^ along the migration path for LaH_3-2*x*_O_*x*_ at *x* = 0.25. The disordering of H^−^ at the Oc site is also shown schematically. **b** Anharmonic oscillation of H^−^ in LaH_3_. Left figure represents the anharmonic oscillator potential of light and highly polarizable H−, and right shows the crystal structure of LaH_3_ where the rhombic dodecahedrons of LaH_14_ are emphasized. The light mass and high polarizability of H^−^, and the framework structure composed of densely packed H^−^ in LaH_3−2*x*_O_*x*_, are crucial factors that impose anharmonicity

The difference in LaH_3−2*x*_O_*x*_ activation energy between experiment and theory is ascribed to oxygen-doping effects; namely, an additional contribution of association enthalpy (*H*_assoc_) to the apparent migration enthalpy, *H*_m_ (Fig. 5a). For LaH_3−2*x*_O_*x*_, the lanthanum bonded to oxygen should bear a larger positive charge, and strongly trap the neighboring H^−^ at the T_D2_ site (which is located adjacent to oxygen, see Fig. 1b). Indeed, the calculations of activation energy for H^−^ hopping from the Oc to the T_D2_ site were unsuccessful because H^−^ positioned at the Oc site moves spontaneously to the T_D2_ site during structural relaxation, which indicates that the H^−^ at the T_D2_ site hardly moves from its initial position (see Supplementary Figs. 7–9).

We now discuss the origin of the large pre-exponential factor *A* at *x* = 0.24. In random-walk theory, the value of *A* is determined by the concentration of mobile ions or vacancies, jump distance, jump frequency, and some physical constants. Thus, we can roughly calculate the value of *A* for LaH_3−2*x*_O_*x*_ by using 3–2*x*, √3/4*c* Å, and 10^15^ s^−1^ for the concentration of mobile ions, jump distance, and jump frequency, respectively^4^. The calculated value falls in the range of ~10^7^ S cm^−1^ K irrespective of *x*, which drastically underestimates the experimental values of 7.8 × 10^11^ S cm^−1 ^K at *x* = 0.24 (see Supplementary Note 5 for details).

Note that the association enthalpy *H*_assoc_ may plausibly depend strongly on temperature. If so, *H*_assoc_ can be equated to *a*−*bT*, where *a* and *b* are constants, in which case the exponential term *E*_a_^exp^(*T*) = *H*_m_ + *H*_assoc_ in Eq. (1) would take the form

2 $${\sigma _{\mathrm{i}}T{\mathrm{ = }}A{\mathrm{exp}}\left( {b{\mathrm{/}}k_{\mathrm{B}}} \right){\mathrm{exp}}\left[ {{\mathrm{ - }}\left( {H_{\mathrm{m}}{\mathrm{ + }}a} \right){\mathrm{/}}k_{\mathrm{B}}T} \right]{\mathrm{ = }}A\prime {\mathrm{exp}}\left[ {{\mathrm{ - }}\left( {H_{\mathrm{m}}{\mathrm{ + }}H_{{\mathrm{assoc0}}}} \right){\mathrm{/}}k_{\mathrm{B}}T} \right]{\mathrm{,}}}$$

where we have replaced *a* with *H*_assoc_ and *A*exp(*b*/*k*_B_) with *A*′. Because *H*_assoc_ is expected to become shallower upon warming due to the anharmonic vibration of ions, the parameter *b* should be positive. Consequently, *A*′ = *A*exp(*b*/*k*_B_) is an exponentially increasing function of *b* and is apparently greater than *A*.

Given the unique features of H^−^ (i.e., light mass and large electronic polarizability), this treatment is probable for LaH_3−2*x*_O_*x*_ with *x* ~ 0 (the left panel in Fig. 5b). Cleary, higher-order terms in the potential energy become increasingly important with increasing atomic displacements. Because the small mass of H^−^ gives rise to large-amplitude vibrations, the anharmonic effect may naturally be considered non-negligible, especially upon warming. A large zero-point energy arising from the light mass also leads to large-amplitude vibrations in the quantum regime. Moreover, the large electronic polarizability of H^−^ also contributes to the anharmonicity of the vibration. During the vibration, the positively charged core of the highly polarizable ion also vibrates inside the strongly deformed electron cloud. This vibration of the core in the electron cloud correlates with the normal harmonic vibration, which adds to the anharmonicity.

In addition to the unique features of H^−^, the vibrations of H^−^ would also be affected by the neighboring H^−^ at *x* ~ 0. At *x* ~ 0, the nearest neighbor of H^−^ trapped at the T_D2_ site is not La^3+^ but H^−^ at the Oc_D4_ site, so the repulsive Coulomb force from the neighboring H^−^ ions modulates the H^−^ vibration and imposes anharmonicity. Moreover, the densely packed H^−^ ions constitute a framework structure with short H^−^–H^−^ separation (the right panel in Fig. 5b). Such a soft lattice with large polarizability reduces the frequencies of the vibrational modes, which, in turn, gives rise to more scattering channels for phonons^18^. Within the quasi-harmonic approximation, the value of the parameter *b* is determined by the product of the thermal expansion coefficient and the Grüneisen parameter which is a measure of anharmonicity (see Supplementary Note 5 for details)^19,20^. Since the squared Grüneisen parameter is proportional to the phonon-phonon scattering probability, the lattice softness originating from the framework structure of H^−^ increases the Grüneisen parameter and the thermal expansion coefficient^21,22^. As a result, a large value is obtained for *b*. Indeed, the Grüneisen parameter of isostructural CeH_3_ is extraordinarily large, ~8 for the acoustic branch and ~4 for the optical branch^23^. The former value exceeds that of state-of-the art thermoelectric materials such as SnSe and PbTe^24^.

As *x* increases, however, the Coulomb repulsion between neighboring H^−^ ions weakens because the inter-H^−^ distance increases. The decoupling of H^−^ vibrations reduces the anharmonicity of vibration of the trapped H^−^ and explains why the pre-exponential factor decreases strongly with *x*. In the same manner, the small pre-exponential factor of BaH_2_ and La_0.6_Sr_1.4_LiH_1.6_O_2_ may also be attributed to the large separation of H^−^ ions in those materials.

By using NPD, XRD, TDS, AC impedance spectroscopy, DC polarization, and first-principles MD simulations, we examine herein the crystal structure, chemical composition, and H^−^ conduction of synthesized oxygen-doped LaH_3_ (LaH_3−2*x*_O_*x*_). The NPD performed on samples with *x*_nom._ = 0.5 and 0.75 indicate that the structure crystallizes in tetragonal with the space group of *P*4/*nmm*, which is a subgroup of fcc structure (i.e., the similar structure to fcc-LaH_3_). Analyses of the chemical composition indicate that, upon oxygen doping into the T site, H^−^ vacancies occur mainly at the Oc site and slightly at the T site. The optimized ionic conductivity of 2.6 × 10^−2 ^S cm^−1^ and the high transport number (>0.99) of ionic (hydride) conduction occur at *T* = 340 °C and at *x* = 0.24. At these intermediate temperatures, this conductivity is the highest reported to date among hydride-ion conductors. Upon increasing the oxygen content *x*, the relatively high activation energy of 1.2–1.3 eV remains almost unchanged, whereas the pre-exponential factor decreases dramatically from the extraordinarily large value of 10^12^ S cm^−1^ K at *x* = 0.24 to 10^8^ at *x* = 0.99, indicating that the pre-exponential factor is the main factor determining the high ionic conductivity and its dependence on oxygen content *x*. In MD simulations with *x* = 0.25, we monitored the mobile and immobile H^−^ ions, the latter of which are trapped by the highly charged lanthanum ion bonded to oxygen. The unusually large pre-exponential factor is a consequence of the significant temperature dependence of association enthalpy for the trapped H^−^. We conclude that the light mass and high polarizability of H^−^, and the structural framework composed of densely packed H^−^ in LaH_3−2*x*_O_*x*_, are crucial factors that impose the temperature dependence on the potential energy, and lead to the characteristic fast H^−^ conduction, i.e., the fast H^−^ conduction controlled by the pre-exponential factor.

## Methods

### Synthesis

Polycrystalline LaH_3−2*x*_O_*x*_ was synthesized by using La_2_O_3_ and LaH_3_ as precursor:

3 $$(3 - 2x)/3\,{\mathrm{LaH}}_3 + x/3\,{\mathrm{La}}_2{\mathrm{O}}_3 \to {\mathrm{LaH}}_{3 - 2x}{\mathrm{O}}_x.$$

The LaH_3_ was prepared by heating La metal at 400 °C for 10 h in a H_2_ atmosphere at 2 MPa partial pressure, and the La_2_O_3_ was used after dehydration by heating to 1200 °C and maintaining that temperature for 2 h. The two precursors were mixed in a dehydrated glass mortar and pressed to form pellets (6 mm diameter and 1–2 mm thick), and then put into a sample cell for a belt-type high-pressure apparatus. Lithium aluminum hydride (LiAlH_4_) as a solid hydrogen source was pelletized in the same shape as the sample and placed above and below the sample pellet. During the synthesis, these hydrogen sources decomposed and provided hydrogen gas. The final product was obtained by heating to 800 °C under a pressure of 750 MPa for 0.5 h^25,26^.

### Analyses of structural and chemical compositions

The crystal structure was analyzed by combining XRD and NPD done by using a Bruker AXS D8 ADVANCE-TXS using a CuKα radiated from a rotational anode and a High-Intensity Total Diffractometer (NOVA) installed at BL21 of the MLF pulsed neutron source in the Japan Proton Accelerator Research Complex, respectively. For the NPD measurement, we prepared deuterated samples LaD_3−2*x*_O_*x*_ with *x*_nom._ = 0.5 and 0.75. The neutron scattering from 6 mm i.d. vanadium rod, empty cell and instrumental background were measured in advance for sample measurements. Observed scattering intensities from samples were corrected for instrumental background, absorption and scattering of sample and cell, detection efficiency of ^3^He position sensitive proportional counters, multiple and incoherent scatterings^27,28^. Rietveld analyses for the diffraction patterns were done by using TOPAS (Bruker AXS, version 4.2) and FullProf.2k (version 6.00) software with the XRD and NPD patterns, respectively^29,30^. We employed a fundamental parameter method implemented in TOPAS to fit the peak profile of XRD, while a pseudo-Voigt function with a pair of back-to-back exponentials implemented in FullProf for NPD data^30–32^. For the NPD data, the measured time of flight (TOF) was converted to *d* value by using the formula: TOF = ZERO + Dtt1 × *d* + Dtt2 × *d*^2^. The three constants were calculated using NPD data of Si standard sample (*a* = 5.431230 Å).

The hydrogen content of samples was measured by using TDS (ESCO TDS-1400TV). A powdered samples placed on a SiO_2_ holder was heated up to 1200 °C, and desorbed hydrogen ions (*m*/*z* = 2) were counted as an electronic current by the quadrupole mass spectrometer. The number of hydrogen molecules was calculated based on the area of the desorption peak corrected by using a silicon wafer containing implanted hydrogen. The chemical composition of LaH_3−2*x*_O_*x*_ was determined from the amount of hydrogen by assuming that each ion has a formal charge (+3, −2, and −1 for La, O, and H, respectively) and that the charge-neutrality condition is satisfied. Peak separation for the observed TDS profile was conducted by using the peak function derived from the general-order kinetics model for thermal desorption, which gives

4 $$I\left( T \right) = ke^{ - \frac{\varepsilon }{T}}\left( {N^p - kp\mathop {\smallint }\limits_0^T e^{ - \frac{\varepsilon }{\tau }}d\tau } \right)^{\frac{1}{p} - 1},$$

where *I*(*T*) is the intensity at temperature *T* and *k*, *ε*, *N*, and *p* are fitting parameters that correspond to the rate constant, activation energy, number of total molecules, and kinetic order of desorption, respectively^33^.

### Conductivity measurements

Pd and Au electrodes were used as a hydrogen-reversible and blocking electrodes, respectively. First, we dropped acetone on PdCl_2_ or AuCl_2_ powder, and then mixed to make suspensions. To dissolve PdCl_2_ in acetone, a small amount of LiCl was added. Next, the suspension was painted onto both sides of the sample, which was then dried at room temperature in an Ar-filled glovebox. Finally, the suspension of metal chloride was reduced and deposited onto the sample surface by heating at 300 °C in a 0.2 MPa H_2_ atmosphere. The painting of metal chloride suspension and heating were repeated twice to ensure the coverage of metal electrode on the pellet surface. The scanning electron microscopy (SEM) images of electrodes are shown in Supplementary Figs. 11–14.

The conductivity of LaH_3−2*x*_O_*x*_ was measured by using AC impedance spectroscopy (Keysight technologies E4990A, frequency: 20 Hz to 10 MHz, voltage amplitude: 500 mV). The Pd was used as a reversible electrode to decrease the interface resistance between sample and electrode. After removing the Pd electrodes, the DC polarization measurement was done with Au electrodes attached. The apparatus for the AC and DC measurements consists of stainless steel (the schematic of apparatus we used is shown in Supplementary Fig. 15), and the electrodes are mechanically attached to the Pd or Au electrode deposited on the sample. An electric current was supplied and recorded by a DC voltage current source and monitor (ADC 6243) in 1 s intervals by applying a constant voltage for 10 min. The applied voltage was varied from 0 to 0.1 V in increments of 0.02 V. Impedance spectroscopy and polarization measurements were both done in the two-wire configuration at temperatures ranging from 100 to 350 °C under Ar flow with a flow rate of 200 mL min^−1^. The temperature dependence of the impedance spectroscopy was performed during heating process.

### First-principles molecular dynamics simulation

The molecular dynamics simulation was followed by structural relaxation, which was done by using density-functional theory implemented in VASP^34,35^. We used the generalized-gradient Perdew–Burke–Ernzerhof functional and the core electrons were described by using the projector-augmented-wave method^36,37^. The unit cell for LaH_3-2*x*_O_*x*_ (*x* = 0.25) is 4[La_4_OH_10_] and includes four vacancy sites, and the 5*s*^2^, 5*p*^6^, 6*s*^2^, and 5*d*^1^ electrons for La, 2*s*^2^ and 2*p*^4^ electrons for O, and 1*s*^1^ electron for H were handled as valence electrons. We adopted the Monkhorst–Pack *k*-point grid of 6 × 6 × 4 and a cutoff energy of 520 eV. The optimized lattice parameters were *a* = 7.975451 Å and *c* = 11.24889 Å, which are within 1.5% of the experimental value of 2*a* and 2*c* at *x*_nom._ = 0.25, respectively.

First-principles MD calculations were also done by using the VASP code with the same density functional and the same parameters for the Monkhorst–Pack *k*-point grid and cutoff energy used in the structural relaxation. The lattice size for the MD simulation is fixed to the one that was optimized in the structural relaxation (i.e., NVT ensemble). We adopted the Nosé–Hoover thermostat to maintain the temperature of 400 °C and ran the MD simulation for 25 ps with 1.0 fs time steps.

The MSD of H^−^/O^2−^ was calculated by using $\frac{1}{N}\mathop {\sum }\limits_{i = 1}^N \left[ {r_i\left( t \right) - r_i\left( 0 \right)} \right]^2$, where *N* is the total number of H^−^/O^2−^ in the unit cell, and *r*_*i*_(*t*) is the position of the *i*th H^−^/O^2−^ at time *t*. The self-diffusion coefficient *D* is calculated as the MSD divided by 6*t* assuming a three-dimensional random walk, and Einstein’s relationship leads to a mobility of the form *μ* *=* *De/k*_B_*T*, where *T* is the temperature and *k*_B_ is the Boltzmann constant. Finally, we obtain a conductivity *σ* = *μnQ*, where *n* and *Q* are the concentration and electric charge of the carrier, respectively.

## Supplementary information

Supplementary Information

## Data Availability

The data that support the plots within this paper and other findings of this study are available from the corresponding authors on request.
