# Supplementary material for: Characteristic fast H− ion conduction in oxygen-substituted lanthanum hydride
Source: Nat Commun. 2019 Jun 12;10:2578. doi: 10.1038/s41467-019-10492-7 (PMC6561957; doi:10.1038/s41467-019-10492-7)
Supplement: Supplementary file 1 — Supplementary Information [file 41467_2019_10492_MOESM1_ESM.pdf]

Supplementary material / Characteristic Fast H^- Ion Conduction in Oxygen-Substituted Lanthanum Hydride

Keiga Fukui[1], Soshi Iimura[1,†,*], Tomofumi Tada[2], Satoru Fujitsu[2], Masato Sasase[2], Hiromu Tamatsukuri[3], Takashi Honda[3, 4], Kazutaka Ikeda[3, 4], Toshiya Otomo[3, 4] and Hideo Hosono[1, 2, ‡,*]

[1]Laboratory for Materials and Structures, Tokyo Institute of Technology, Yokohama 226-8503, Japan

[2]Materials Research Center for Element Strategy, Tokyo Institute of Technology, Yokohama 226-8503, Japan

[3]Institute of Materials Structure Science, High Energy Accelerator Research Organization (KEK), Tsukuba 305-0801, Japan

[4]Department of Materials Structure Science, The Graduate University for Advanced Studies, Tsukuba 305-0801, Japan

Correspondence and requests for materials should be addressed to S. Iimura and H. Hosono.

[*,†] S. Iimura, e-mail: s_iimura@mces.titech.ac.jp

[*,‡] H. Hosono, e-mail: hosono@msl.titech.ac.jp

Supplementary Note 1

For the NPD, first we refined the crystal structure of $LaD_2O_{0.5}$ by using a space group of *Fm*-3*m* (fcc structure) in which the oxygen is disordered at T-site and the off-centering of D at O-site is considered. However, the reliable factor for the fitting, *Rwp* = 17.5% and χ^2 = 8.24, were not good, and the net charge neutrality between La^{3+}, O^{2-} and D^- was not preserved in the refined structure. Then, next we considered an oxygen-ordering that frequently observed in rare earth oxyfluorides. The crystal structure of oxyfluorides, so called Vernier-phase, crystallizes in a pseudo-fluorite structure with a tetragonal or orthorhombic distortion.[1] Among those complex structures, we adopted a tetragonal structure with a space group of *P*4/*nmm* as the simplest model with the smallest lattice constants.[2] This model successfully reduced the reliable factors to *Rwp* = 5.93% and χ^2 = 7.31 and some of small peaks which were not indexed when using *Fm*-3*m* can be indexed (Supplementary Figure 1a, b), and the refined chemical composition agreed well with that measured by TDS. We also performed the NPD for $LaD_{1.5}O_{0.75}$ and analyzed using same structure model as used at $x_{\mathrm{nom.}}$ = 0.5. The profile is shown in Supplementary Figures 1c and d. In Supplementary Table 1, we summarized the structural parameters refined using the tetragonal *P*4/*nmm* at $x_{\mathrm{nom.}}$ = 0.5 and 0.75.

In order to know an accurate space group, we carried out the electron diffraction measurement on $LaH_2O_{0.5}$, and pursued an additional spots derived from the incommensurate structure. However, we only observed spots derived from the fcc structure as shown in Supplementary Figure 2, suggesting that oxygens as well as hydrogens are ordered in quite long range and that the model *P*4/*nmm* is insufficient for fully accounting the range of oxygen ordering. Combing results of neutron, x-ray and

electron diffractions, the lanthanum forms the fcc structure, but anions are ordered in quite long range as observed in the Vernier-phases.[1]

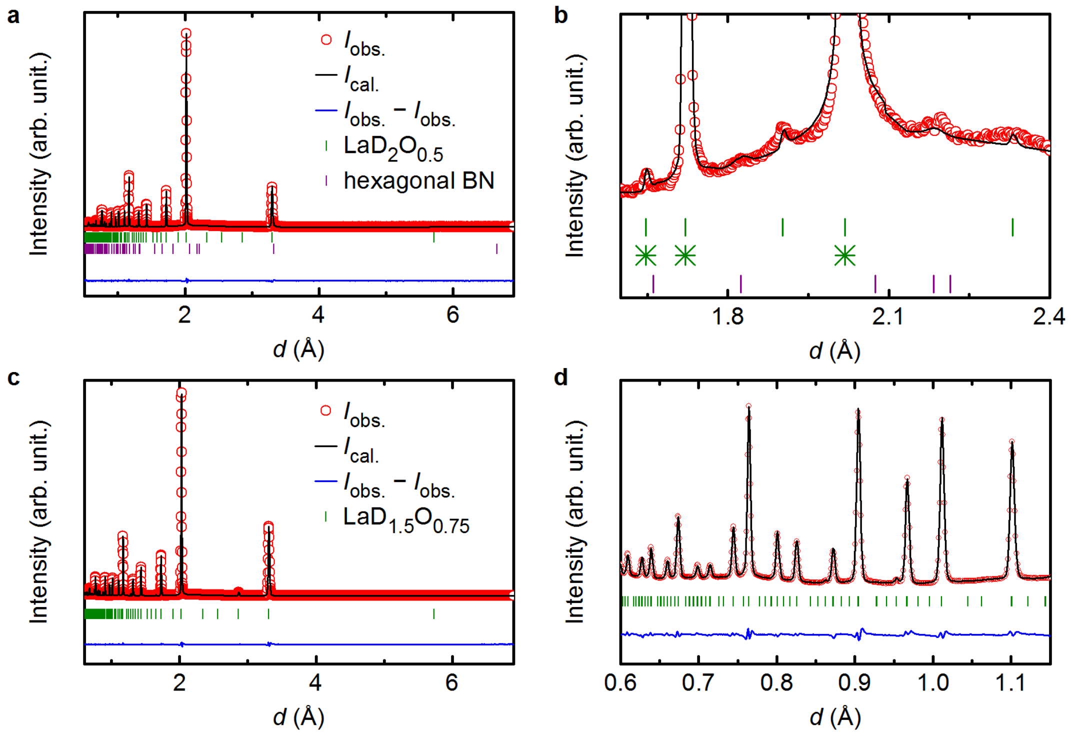

Supplementary Figure 1 Neutron diffraction patterns of $LaD_2O_{0.5}$ and $LaD_{1.5}O_{0.75}$ ($x_{nom.}$ = 0.5 and 0.75, respectively) at room temperature. **a, b** NPD patterns used for the Rietveld analysis (**a**) and an expanded view at *d* ~ 2Å. (**b**). Red circle and black solid line denote observed and calculated intensities, respectively, and the blue solid line is a difference between them. The green and purple bars below the diffraction patterns represent diffraction positions of $LaD_2O_{0.5}$ with *P*4/*nmm* and hexagonal BN. In Supplementary Figure 1b, diffraction positions of $LaD_2O_{0.5}$ with *Fm*-3*m* structure is also shown by asterisks. **c, d** NPD patterns used for the Rietveld analysis (**c**) and the expanded view in the *d* range from 0.60 to 1.15Å. (**d**). The green bars below the diffraction patterns represent diffraction positions of $LaD_{1.5}O_{0.75}$ with *P*4/*nmm*.

Supplementary Table 1 Crystallographic data of $LaD_2O_{0.5}$ and $LaD_{1.5}O_{0.75}$. Structural parameters of $LaD_2O_{0.5}$ and $LaD_{1.5}O_{0.75}$. The space group is *P*4/*nmm* with an origin choice of 2, and the refined lattice parameters are $a = b = 4.0338(5)$ Å and $c = 5.703(1)$Å for $LaD_2O_{0.5}$ and $a = b = 4.0438(3)$ Å and $c = 5.7172(8)$Å for $LaD_{1.5}O_{0.75}$. The reliable factors were calculated to be *Rexp* = 2.19%, *Rwp* = 5.93% and $\chi^2 = 7.31$ for $LaD_2O_{0.5}$, and *Rexp* = 0.835%, *Rwp* = 6.22% and $\chi^2 = 55.58$ for $LaD_{1.5}O_{0.75}$. The higher χ^2 of $LaD_{1.5}O_{0.75}$ than that of $LaD_2O_{0.5}$ is due to the longer counting time for $LaD_{1.5}O_{0.75}$ than for $LaD_2O_{0.5}$.

$x_{nom.} = 0.5$								
Atom	Symbol	*x*	*y*	*z*	Occ.	*B*	Site	Sym.
La	La1	¼	¼	0.2481(5)	1	0.766(9)	2*c*	4*mm*
O	O1	¾	¼	0	0.66(2)	1.23(2)	2*a*	−4*m*2
D	D1	¾	¼	0	0.343(2)	2.22(2)	2*a*	−4*m*2
D	D2	¾	¼	½	0.812(4)	1.63(3)	2*b*	−4*m*2
D	D3	¼	0.062(4)	0.670(4)	0.1070(8)	2.3(1)	8*i*	.*m*.
D	D4	¼	0.124(6)	0.816(6)	0.0631(9)	2.3(2)	8*i*	.*m*.
$x_{nom.} = 0.75$								
Atom	Symbol	*x*	*y*	*z*	Occ.	*B*	Site	Sym.
La	La1	¼	¼	0.252(1)	1	0.83(2)	2*c*	4*mm*
O	O1	¾	¼	0	0.80(2)	1.2(2)	2*a*	−4*m*2
D	D1	¾	¼	0	0.20(2)	2.22(3)	2*a*	−4*m*2
D	D2	¾	¼	½	0.87(1)	1.31(2)	2*b*	−4*m*2
D	D3	¼	0.052(3)	0.666(3)	0.038(3)	1.7(3)	8*i*	.*m*.
D	D4	¼	0.119(2)	0.817(2)	0.041(2)	1.1(3)	8*i*	.*m*.

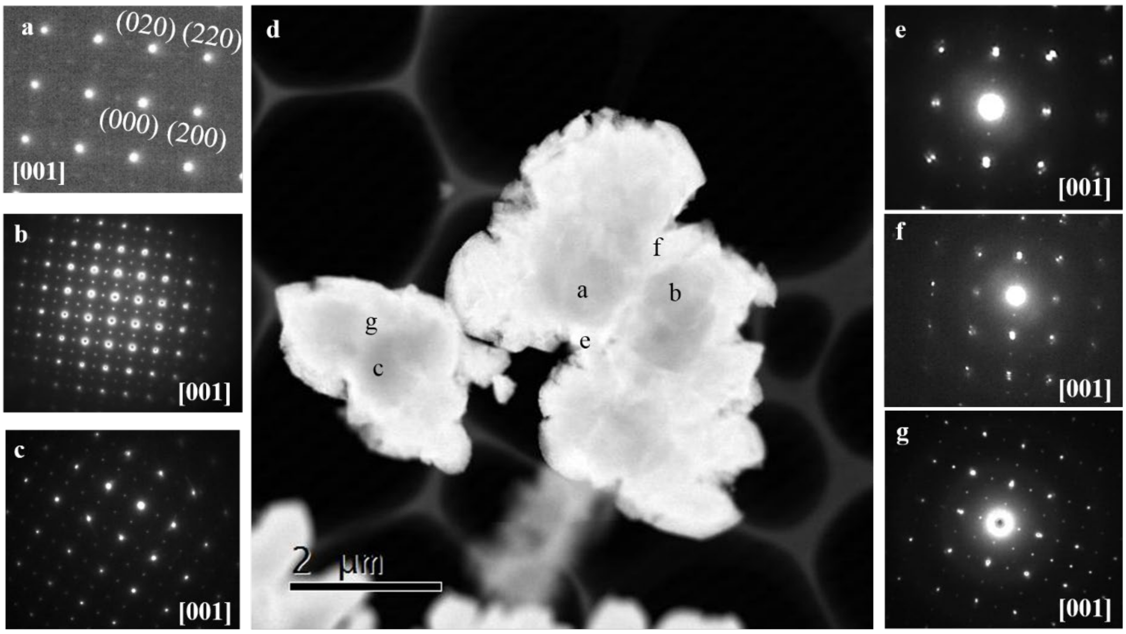

Supplementary Figure 2 Selected-area electron diffraction patterns taken from $LaH_2O_{0.5}$. **a-c**, **e-g**, The diffraction patterns taken from points denoted in Supplementary Figure 2d. The diameter of each area taken those diffraction is around 0.5μm. **d,** High-angle Annular Dark Field Scanning image of $LaH_2O_{0.5}$. **a-c** and **e** were taken from the center of grains, while **f** and **g** were from the grain boundary. Small spots deviated from the fcc lattice are due to a tilting of crystals at the grain-boundary.

Supplementary Note 2

To examine the effect of grain boundary on the ionic conductivity, we newly synthesized the sample with $x_{\mathrm{nom.}} = 0.25$ by using a new sample cell for the high pressure synthesis. The constituent material of sample cell sometime affects the sintering of sample, i.e., the grain boundary and relative density, because the isotropy of pressure applied to sample is sensitive to the material used for the sample cell. The conventional and new sample cell assemblies are shown in Supplementary Figure 3a and 3b, respectively. We partly replaced the NaCl tube around the sample with the hexagonal boron nitride (hBN) tube.

Then, we synthesized the sample at the same temperature and pressure as described in main text, and took the SEM images and conductivity data. Supplementary Figure 4 compares the SEM images of samples at $x_{\mathrm{nom.}} = 0.25$ prepared using the different sample cells, NaCl for S4a-c and hBN for S4d-f. There are some voids and each grain is observed in the sample prepared using the NaCl cell, while those voids and grain boundary are hard to see in the case of hBN cell. The relative density of the pellets was increased from 86% for the NaCl case to 94% for the hBN case.

Supplementary Figures 5 compare the results of AC impedance spectroscopy and DC polarization measurement on the two samples with $x_{\mathrm{nom.}} = 0.25$. At low temperatures, both samples show a clear half-circle response in the higher frequency region (Supplementary Figures 5a and d). The ionic conductivities evaluated from the half-circle ($R1$) are very close, that is, 4.1×10^{-5} and 3.9×10^{-5} $\mathrm{Scm^{-1}}$ for the NaCl- and hBN-cell sample, respectively (see Table S2). On the other hand, the conductivity values from $R2$ corresponding to the lower frequency region were 6.5×10^{-6} $\mathrm{Scm^{-1}}$ and 1.2×10^{-5}

Scm^{-1} for the NaCl- and hBN-cell sample, respectively. This result means that the ionic conductivity of bulk (calculated from $R1$) remains unchanged, while that of grain boundary (calculated from $R2$) is improved due to the higher relative density of the hBN-cell sample. Supplementary Figures 5b and e show the AC impedance spectrum taken at high temperatures. Although the half-circle response becomes vague, the intercept of x axis, which corresponds to the resistance of the bulk, are close each other (see Supplementary Figure 5f). The results of DC polarization measurements on both the samples are shown in Supplementary Figures 5c and g. In the case of hBN cell sample, the electronic conductivity estimated from the steady-state current was $\sigma_e = 9.34 \times 10^{-5}$ S cm^{-1} at $T = 337$°C, which also is similar with $\sigma_e = 4.85 \times 10^{-5}$ S cm^{-1} of the NaCl cell sample. The transport number of the hBN cell sample at $T = 340$°C was evaluated to 0.998 which is the same as that of the NaCl cell sample.

Supplementary Figure S6 summarizes the conducting properties of samples prepared using the NaCl cell or the hBN cell. The Arrhenius plots of ionic conductivity and x dependences of transport number, activation energy, and prefactor of the sample prepared using the hBN cell are located along the trend observed in the samples prepared using the NaCl cell. Based on those results, we concluded that the primary conductivity observed here does not originate to the grain boundary but bulk of sample.

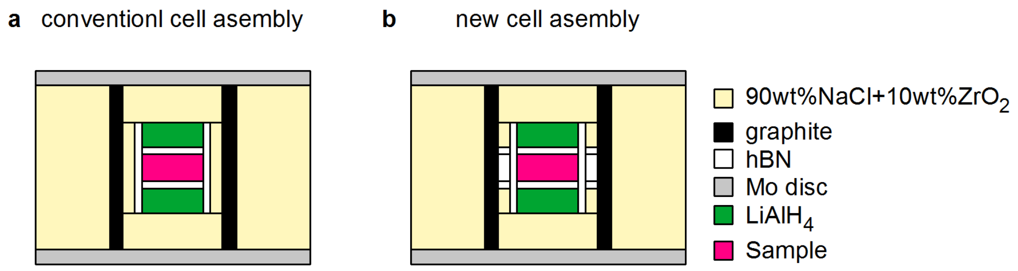

Supplementary Figure 3 Sample cell assemblies for high pressure synthesis of $LaH_{3-2x}O_x$. **a** A conventional sample cell composed of 90 wt%-NaCl and 10 wt%-ZrO_2. The graphite sleeve is used as a resistance heater touching two Mo discs for electric lead below and above the cell. The hBN sleeve of 6 mm (7 mm) in inner (outer) diameter and 8 mm in length is inserted to the NaCl (+10 wt%ZrO_2) tube, and the sample pellet is placed inside the hBN crucible. Two pellets (6 mm in diameter and 2 mm in thickness) of $LiAlH_4$ were placed below and above the sample pellet to supply excess hydrogen through the synthesis. **b** A new sample cell. The NaCl tube around the sample pellet is replaced with hBN tube.

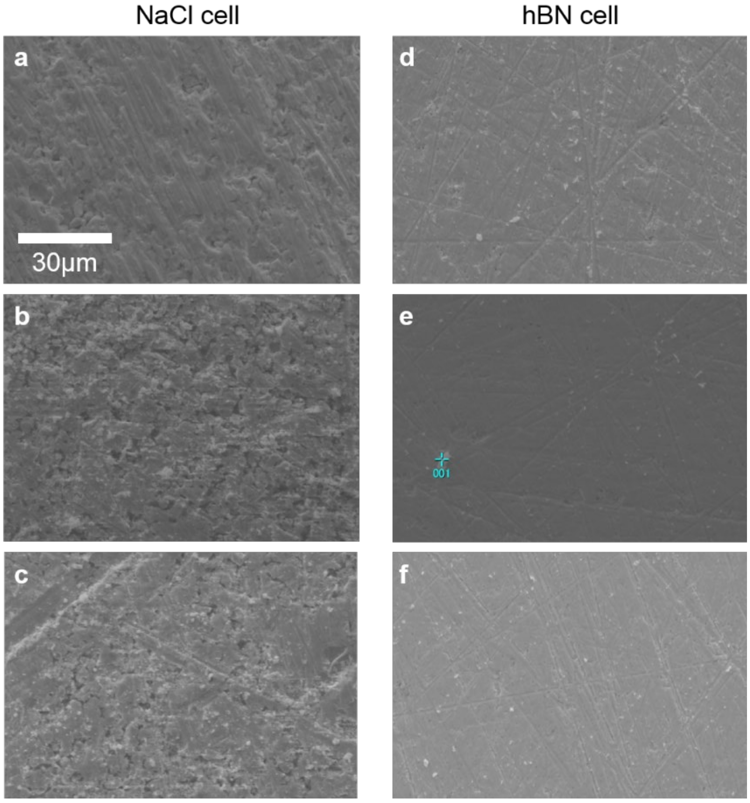

Supplementary Figure 4 Scanning electron microscope images of $LaH_{3-2x}O_x$ with $x_{nom.}$ = 0.25 synthesized using the NaCl or BN cell. $LaH_{3-2x}O_x$ with $x_{nom.}$ = 0.25 prepared using the NaCl (**a-c**) and hBN cells (**d-f**). Each image was taken from the surface perpendicular to the pellet thickness direction. The common scale bar is shown in Supplementary Figure 4a. The relative density of sample prepared using the NaCl and hBN cells are 86% and 94%, respectively.

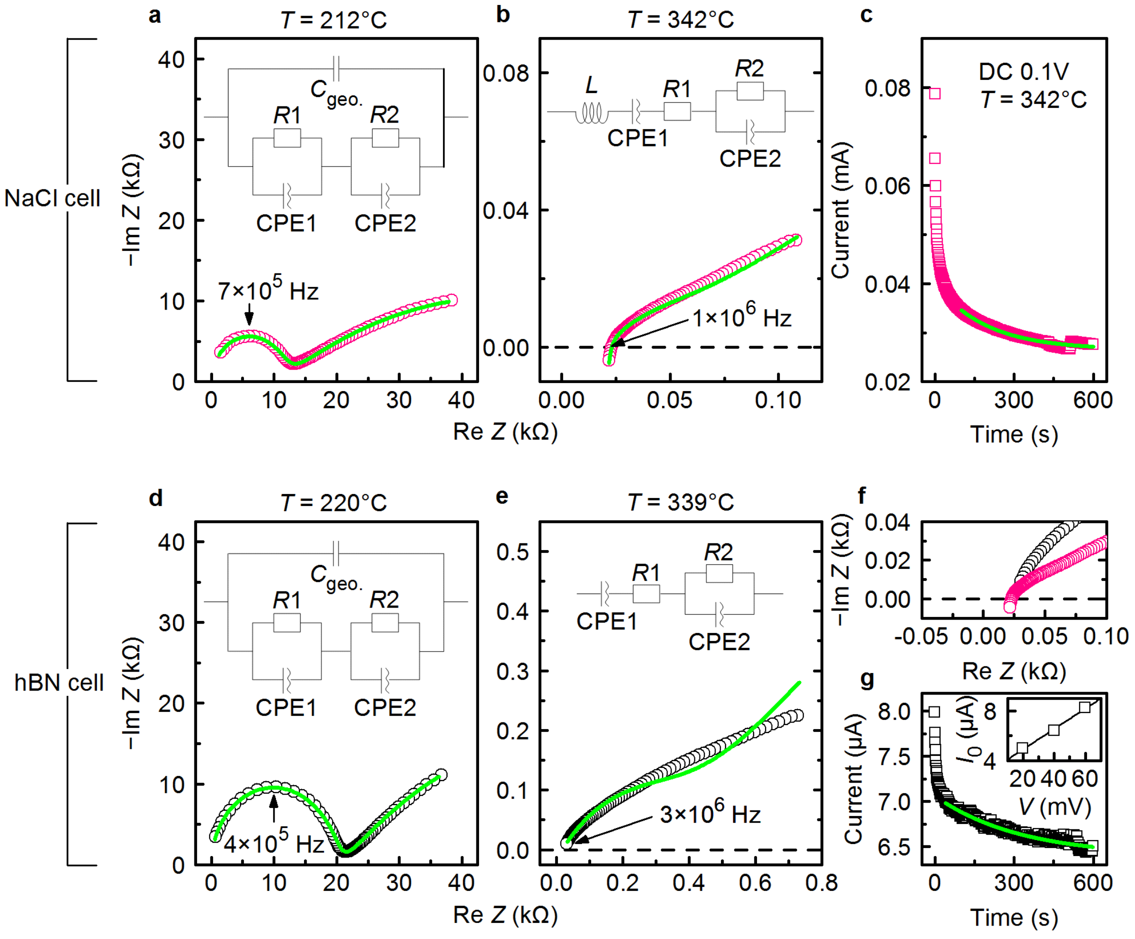

Supplementary Figure 5 Results of AC impedance spectroscopy and DC polarization measurement at $x_{nom.}$ = 0.25 prepared using the conventional NaCl cell or the hBN cell. **a, b, d, e** Complex impedance plots of sample prepared using the conventional NaCl cell (**a, b**) or the hBN cell (**d, e**). The measurement temperatures are shown above the panels. **f** The expanded view of high frequency region at higher temperature. Pink and black open circles denote the complex impedance plots of samples prepared using the conventional NaCl (T = 342°C) and hBN cell (T = 339°C), respectively. **c, g** Current as a function of time upon application of constant voltage. The relaxation current shown in Supplementary Figure 5c was measured at 0.1 V and 342°C, and the data in

Supplementary Figure 5g at 0.04 V and 337°C. In the inset of Supplementary Figure 5g, the steady-state current (I_0) as a function of applied voltage is shown.

Supplementary Table 2 Comparison of *R*1 and *R*2 values. The values of resistance are taken by fitting on the data shown in Supplementary Figure 5a and d.

NaCl cell					hBN cell				
l (cm)	*S* (cm^2)	*R*1 (Ω)	*R*2 (Ω)	Relative density (%)	*l* (cm)	*S* (cm^2)	*R*1 (Ω)	*R*2 (Ω)	Relative density (%)
0.12	0.264	11182	70244	86	0.179	0.205	22131	72345	94

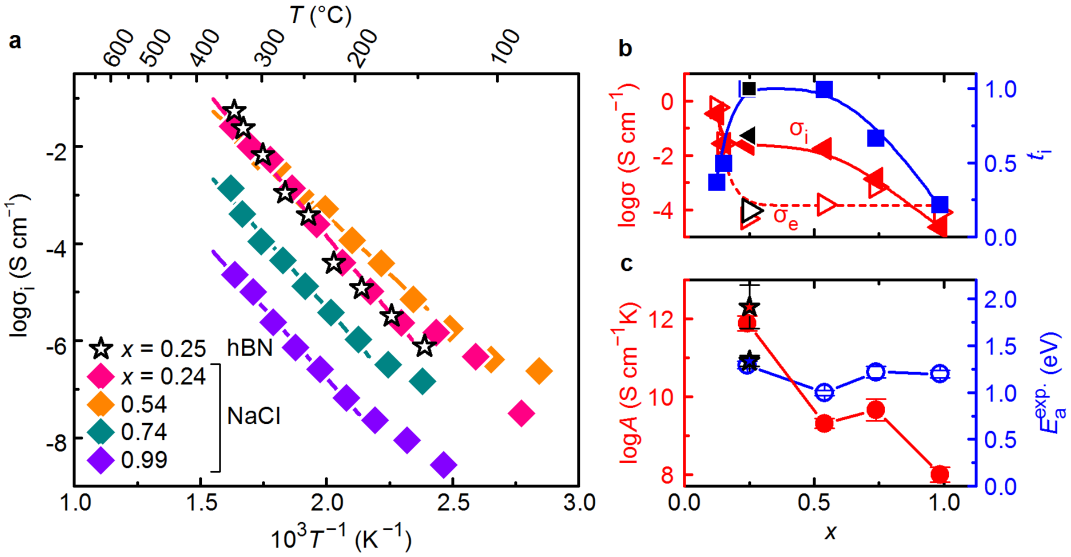

Supplementary Figure 6 x dependence of ionic and electronic conductivities. **a** Arrhenius plots of $LaH_{3-2x}O_x$. Open star symbols are data taken from $LaH_{3-2x}O_x$ with $x_{nom.}$ = 0.25 synthesized using the hBN cell, and other data are from $LaH_{3-2x}O_x$ synthesized using the conventional NaCl cell. **b** Conductivity and ion transport number as functions of oxygen content x at T = 340°C. Red open and solid triangles denote electronic (σ_e) and ionic (σ_i) conductivities, respectively, while blue solid squares are the ion transport number (t_i). Black symbols denotes those of sample prepared using the BN cell. **c** Logarithm of pre-exponential factor A (red solid) and activation energy E_a^{exp} (blue open) as functions of oxygen content x. Black symbols represent those values of sample prepared using the BN cell.

Supplementary Note 3

Activation barrier calculations were executed using the nudged elastic band (NEB) method with climbing image (CI) approach implemented in VASP-DFT code. The force tolerance of CI-NEB is 0.03 eV/Å, and the image number is 10. The other parameters in the calculations are the same to those written in Method section.

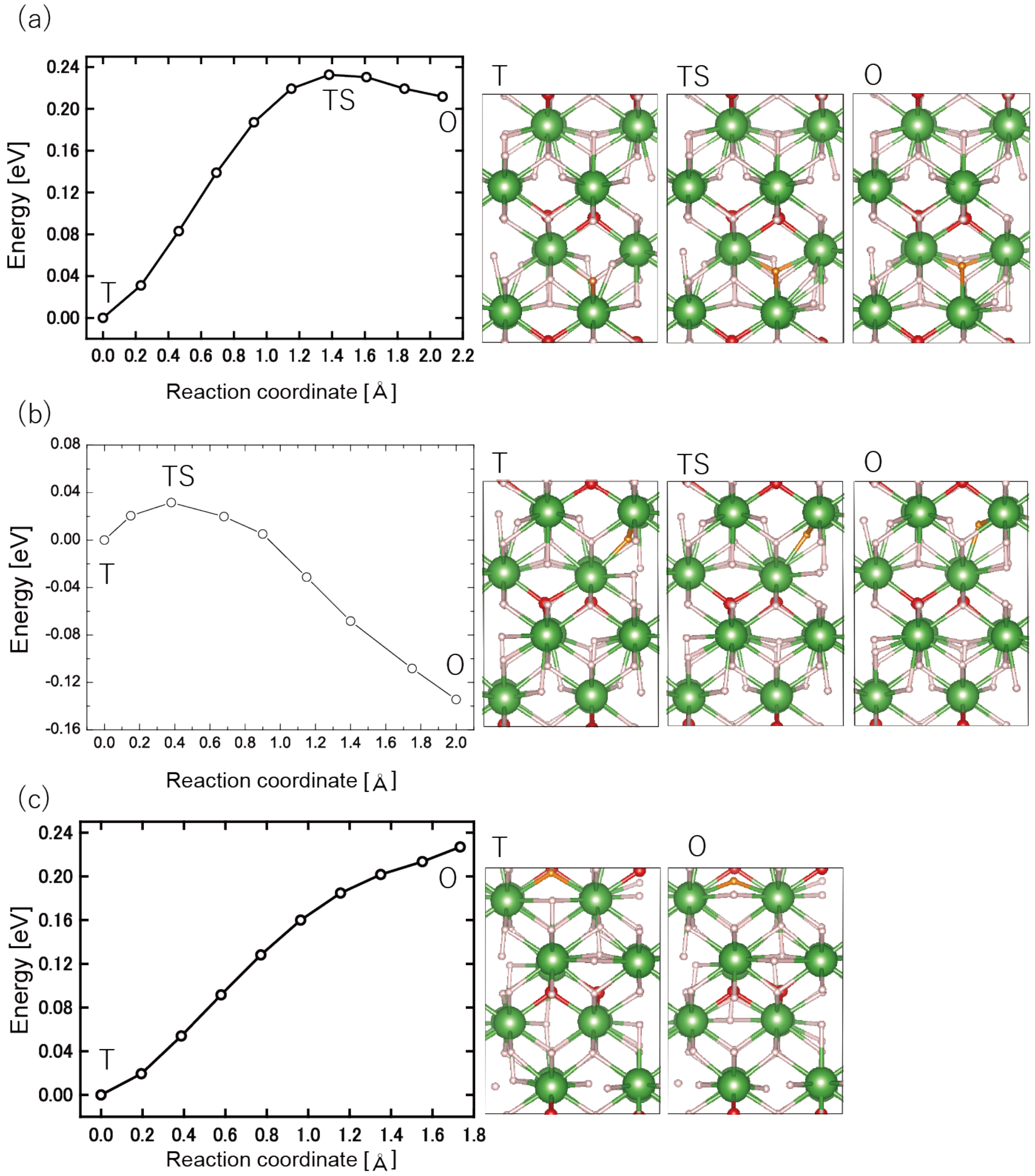

Supplementary Figure 7 Calculated reaction barriers and migration paths of H^- hopping. **a-c** H^- hopping from T_{D1} to Oc_{D3} (**a, b**), and from T_{D2} to Oc_{D4} (**c**). Hopping barriers were confirmed in T_{D1} to Oc_{D3} hopping events, but no barrier in T_{D2} to Oc_{D4} event. The H^- ion to be migrated is depicted with yellow color; La, O, H are depicted with green, red, and white, respectively.

Supplementary Note 4

Supplementary Figure 8 is the histogram that counts the number of entrance and exit of H^{-} at each the tetrahedron or octahedron during MD simulation. The hedron index (ID) from 17 to 24 and from 33 to 40 correspond to the tetrahedra which shares the edge of tetrahedron occupying oxygen. You can see that the hopping event via the tetrahedra with the ID from 17 to 24 and from 33 to 40 is highly limited, suggesting that the activation energy of H^{-} hopping via those tetrahedra is higher than others. Due to the small unit cell size of our structure model that we used for the simulation, the oxygen in the structure is ordered in layer (see the structure model shown in Supplementary Figure 9). This means that our ab initio MD simulation cannot correctly take into account the effect of the large potential barrier hopping around oxygen on the calculated mean square distance and the conductivity. In the H^{-} rich layer, the H^{-} hopping with low activation energy predominantly occurs. Therefore, our simulation is enough to clarify the immobile nature of O^{2-} in the material but insufficient to obtain the quantitative information on H^{-} diffusion barrier via the tetrahedral site which shares the edge of tetrahedron occupying O^{2-}.

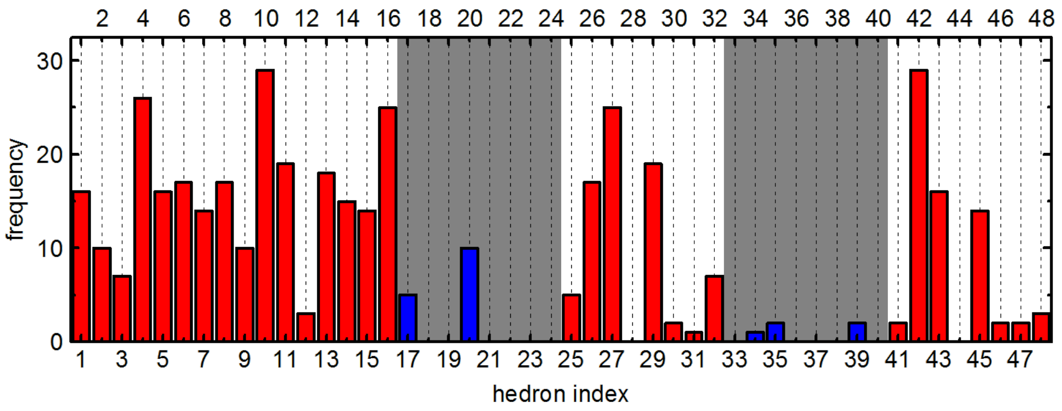

Supplementary Figure 8 Histogram of H^- hopping event at each La4-tetrahedron or La6-octahedron through MD simulation. The frequency of hopping events via the tetrahedra with the ID from 17 to 24 and from 33 to 40 are shaded.

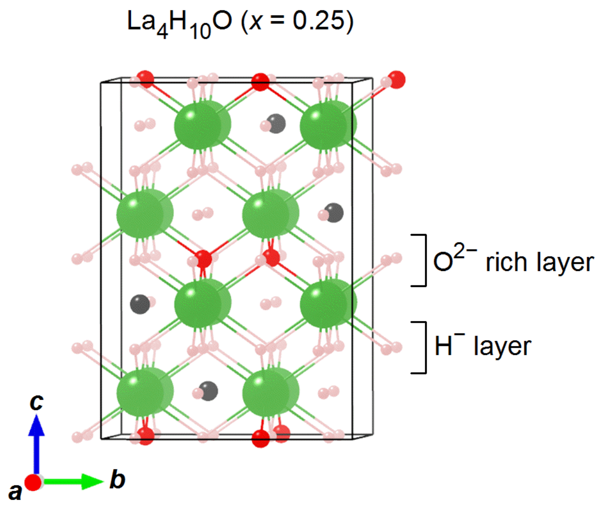

Supplementary Figure 9 Structure of $La_4H_{10}O$ (x = 0.25) used for an initio MD simulation. Green, pink and red spheres represent La, H, and O atoms, respectively. Black sphere denotes the position of vacancy.

Supplementary Note 5

A simplest way to add anharmonicity into harmonic potential is to take into account effects of thermal expansion of bond length on warming and a resulting frequency reduction of elongated bonds.[3] This effect can be treated by assuming that the temperature slope, *b*, corresponds αγ (quasi-harmonic approximation), where α is thermal expansion coefficient and γ is Grüneisen parameter:

$$\gamma = -\frac{dln\omega}{dlnV} \tag{1}$$

where ω and V are vibration frequency of atom and volume, respectively. In this case, the mean square displacement of vibrating atom, $\langle u^2 \rangle$, increases more compared to the harmonic case, as expressed to $\langle u^2 \rangle = k_B T/k\ (1 + 2\alpha\gamma T)$, where the k is force constant.[3]

The Grüneisen parameter γ is a measure of anharmonicity. For the simplest example, we consider an one-dimensional system, *i. e.*, a linear monatomic chain, with only one-type force constant, k (second derivatives of the potential energy) and a length of $L = Na$, where N is the number of atom and a is the equilibrium length of spring. In this case the γ is expressed as $k'a/2k$ in which k' is a third derivatives of the potential energy.[4–6]
harmonic case, as expressed to $\langle u^2 \rangle = k_B T/k\ (1 + 2\alpha\gamma T)$, where the k is force constant.[3]

In the following, we describe how the anharmonicity affects the prefactor. First, we explain the expression of prefactor based on the random walk theory, and then add the anharmonic effect into the prefactor.

The conductivity of charged species is given by

$$\sigma = c/V\ e\ \mu, \tag{2}$$

where c is the number of carrier in unit cell, V is the volume of unit cell per chemical formula, e is the ionic charge, and μ is the mobility of the charge carrier. The mobility is related to the corresponding diffusion coefficient by the Nernst-Einstein relation:

$$\mu = eD/k_BT. \tag{3}$$

The diffusion coefficient, D, is related to its mean jump frequency f by

$$D = (1/6)fd^2 \tag{4}$$

where d is the jump distance. Eq.(4) is based on the random walk theory. Then, f is given by:

$$f = zf_0\exp(-H_m/k_BT), \tag{5}$$

where f_0 is the jump frequency in one specific direction, z is the number of directions in which the jump may occur, H_m is the jump activation enthalpy, and f_0 is given by

$$f_0 = \upsilon_0\exp(S_m/k_B), \tag{6}$$

S_m being the migration entropy and υ_0 an appropriate lattice vibration frequency. Combining eq.s(2)−(6) gives the Arrhenius relation:

$$\sigma T = zd^2e^2c\upsilon_0/6Vk_B\exp(S_m/k_B)\exp(-H_m/k_BT) \tag{7}$$

$$A = zd^2e^2c\upsilon_0/6Vk_B\exp(S_m/k_B) \tag{8}$$

where A is the prefactor in case of random walk theory.

In Table S3 we summarized values of each physical parameter of $LaH_{3-2x}O_x$ described above. Here we considered H^- hopping from tetrahedral site to octahedral site

which is observed in the molecular dynamics simulations. Then, the d corresponds to $\sqrt{3}/4a$ where a is lattice constant of tetragonal $LaH_{3-2x}O_x$. z is 4 because the tetrahedral site is surrounded by four octahedral sites. For the value of υ_0 we used the vibrational frequency of hydrogen that occupies tetrahedral site of LaH_3 (125meV).[7] It is difficult to estimate the migration entropy, so that we referred the S_m/k of alkali halides which are in range from 10 to 60.[8] You can see that the calculated prefactor A seriously underestimates values observed experimentally which are in range from ~10^8 to ~10^{12}.

Supplementary Table 3 Physical constants used to calculate the prefactor based on random walk theory.

x	c	a (Å)	z	d ($\times10^{-8}$ cm)	v_0 ($\times10^{13}$ s^{-1})	$\exp(S_m/k_B)$	V ($\times10^{-23}$cm^3)	A ($\times10^7$Scm^{-1}K)
0.99	1.02	5.720	4	2.477	3.02	35	4.679	1.8
0.74	1.52	5.725	4	2.479	3.02	35	4.692	2.6
0.54	1.92	5.718	4	2.476	3.02	35	4.673	3.3
0.24	2.52	5.703	4	2.469	3.02	35	4.637	4.3

To add the anharmonic effect into the calculated prefactor, we adopt a quasi-harmonic approximation. The quasi harmonic approximation proposed by Wills B. T. M. is to replace the coefficients of second order derivative of potential energy (force constant), k, with the temperature-dependent term, $k_0(1-2\alpha\gamma T)$, where k_0 is force constant at $T = 0$, α is volumetric thermal expansion coefficient, and γ is Grüneisen parameter.[3] Here we consider the temperature dependence of the quasi-harmonic potential energy:

$$U(r, T) = U_0 + k_0(1 - 2\alpha\gamma T)(r - R)^2 \quad (9)$$

$$= [U_0 + k_0(r - R)^2] - 2\alpha\gamma k_0(r - R)^2 T \quad (10)$$

where R is the equilibrium bond length of oscillator, and U_0 is the potential at $r = R$. We set $U_0 = 0$, and consider the potential energy at $r = r'$ in which activation energy barrier is formed. In the following, we assume that the temperature dependency of the quasi-harmonic potential energy can be regarded to be similar with that of activation energy.

In main text, we suggest that H_{assoc} can be equated to $a - bT$, and that the prefactor becomes greater than A by a factor of $\exp(b/k_B)$. If we put r' into r of the eq.(10), and adopt that a corresponds to $k_0(r' - R)^2$ and b does $2\alpha\gamma k_0(r' - R)^2$, the equation (10) is expressed by:

$$U(r', T) = k_0(r' - R)^2 - 2\alpha\gamma k_0(r' - R)^2 T \quad (11)$$

$$= a - bT \quad (12)$$

where b corresponds to $2\alpha\gamma a$.

Now we can quantitatively calculate the enlarged prefactor by using α, γ, and $k_0(r' - R)^2$. In main test, we attributed the large difference in experimental (~1.20eV) and calculated activation energies (~0.1eV) to be $a = H_{assoc0}$ (see equation (2) in page 11). Therefore, $k_0(r' - R)^2 = a$ is estimated to be ~1.10eV or ~$1.10\times1.602\times10^{-19}$ J. For the volumetric thermal expansion coefficient α, we use 3.8×10^{-5} K^{-1} of $LaH_{2.90}$ at 300K, which is almost identical to that of La_2O_3 (3.9×10^{-5} K^{-1}).[9, 10] For the Grüneisen parameter, we use γ of CeH_3 ($\gamma = 8$) for $x_{nom.} = 0.25$ on the assumption that the most sensitive vibrational mode to volume change dominantly contributes to the anharmonicity.[11] In the

ideal harmonic crystal, the γ takes 1, so that we use γ = 1, 3.33, 5.66 for $x_{nom.}$ = 1.0, 0.75, 0.5, respectively. Table S4 summarizes those values as well as the calculated ($A\exp(b/k_B)$) and experimental prefactors (A^{exp}) at each x. Supplementary Figure 10 shows x dependence of A, $A\exp(b/k_B)$, and A^{exp}. The $A\exp(b/k_B)$ that includes the anharmonic effect follows the trend of A^{exp}.

Supplementary Table 4 Physical constants used to calculate the prefactor taking into account the anharmonicity.

x	a ($\times 10^{-19}$ J)	γ	α ($\times 10^{-5}$ K^{-1})	$\exp(b/k_B)$	$A\exp(b/k_B)$ (Scm^{-1}K)	A^{exp} (Scm^{-1}K)
0.99	1.76	1	3.8	2.63	4.66×10^{7}	1.00×10^{7}
0.74	1.79	3.333	3.8	26.80	7.02×10^{8}	4.63×10^{8}
0.54	1.43	5.666	3.8	87.59	2.89×10^{9}	2.05×10^{9}
0.24	1.91	8	3.8	4513.64	1.96×10^{11}	7.78×10^{11}

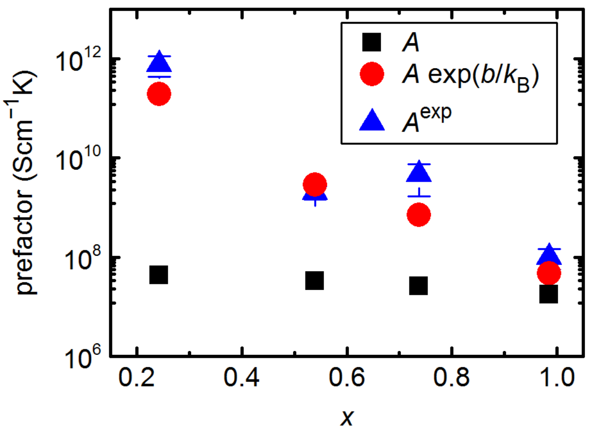

Supplementary Figure 10 x dependence of the calculated prefactor A, $A\exp(b/k_B)$, and experimental A^{exp}.

Supplementary Note 6

Supplementary Figure 11 is the cross-sectional SEM image of Pd electrode deposited on sample at $x_{nom.} = 0.25$. From this figure, the thickness of Pd electrode is estimated to be 22 μm. Supplementary Figure 11a is the top-view of the Pd electrode. The Pd electrode covers homogeneously the surface of pellet, which is obvious if you compare it with the SEM image of Pd electrode deposited only once shown in Supplementary Figure 12b, where La and O are exposed at the void of Pd electrode. The cross-sectional images and top-view of Au electrode are shown in Supplementary Figures 13 and 14, respectively. The thickness of Au electrode was estimated to be 6 μm from Supplementary Figure 13.

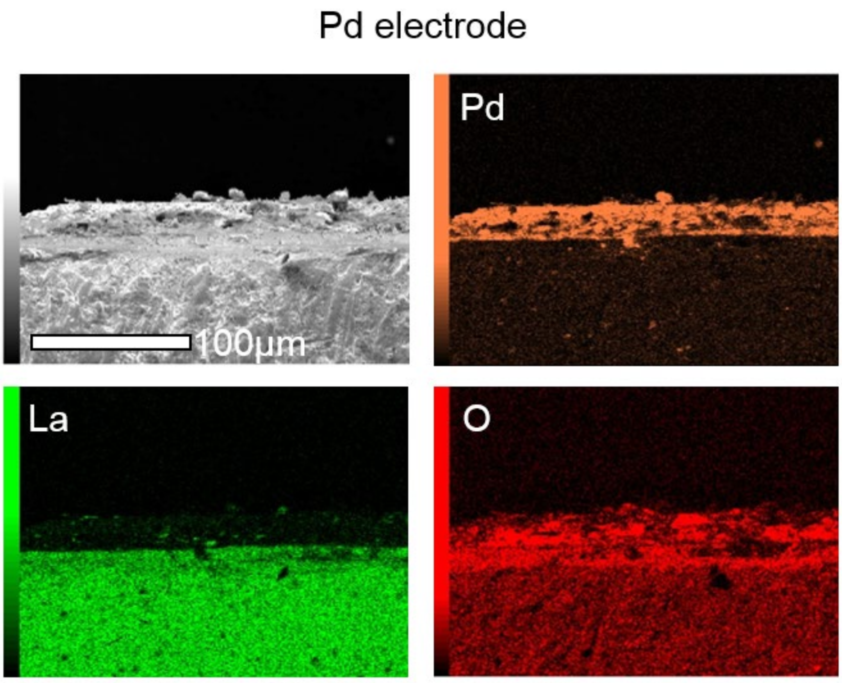

Supplementary Figure 11 Results of EDX mapping on the cross section of Pd-deposited $LaH_{3-2x}O_x$ with $x = 0.25$. The images were taken after the AC impedance measurements.

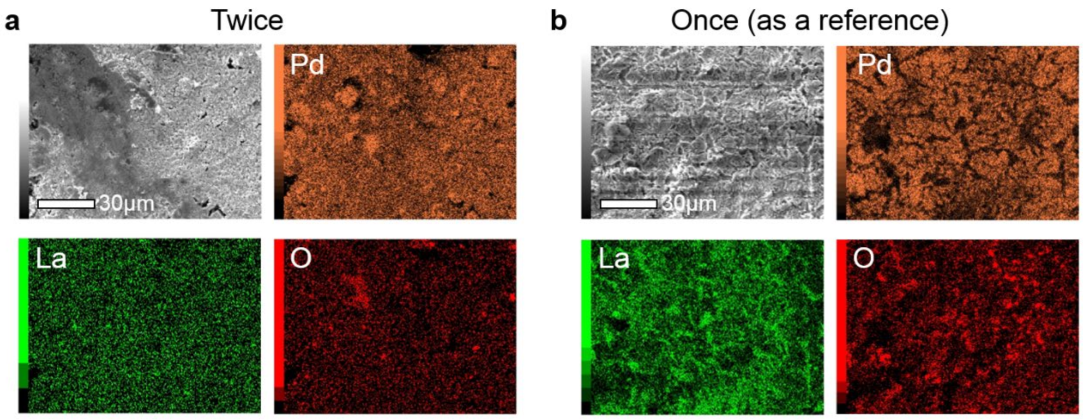

Supplementary Figure 12 Results of EDX mapping on the surface of Pd electrode deposited on $LaH_{3-2x}O_x$ with $x_{nom.} = 0.25$. The images were taken after the AC impedance measurements.

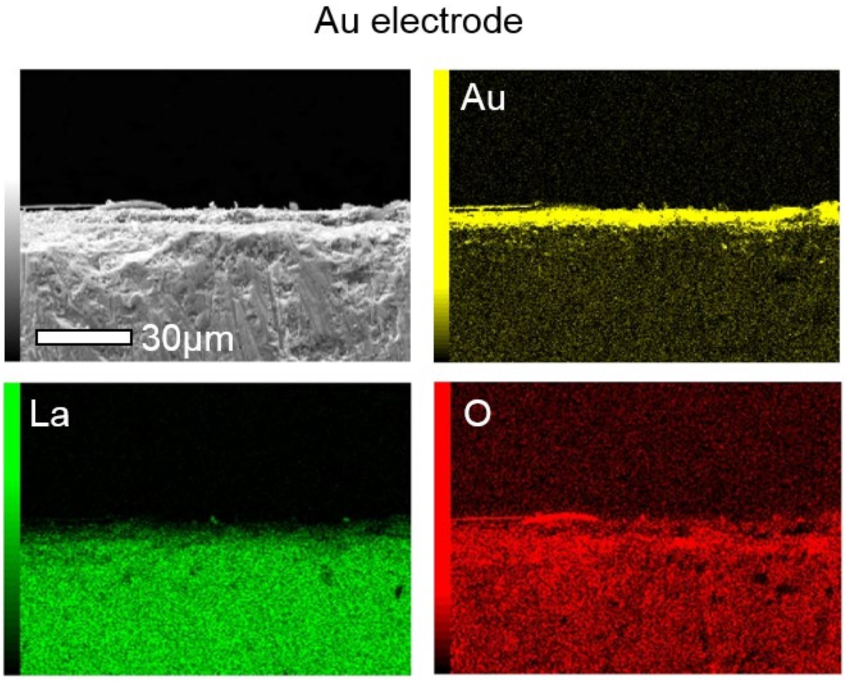

Supplementary Figure 13 Results of EDX mapping on the cross section of Au-deposited $LaH_{3-2x}O_x$ with $x = 0.25$. The images were taken after the DC polarization measurements.

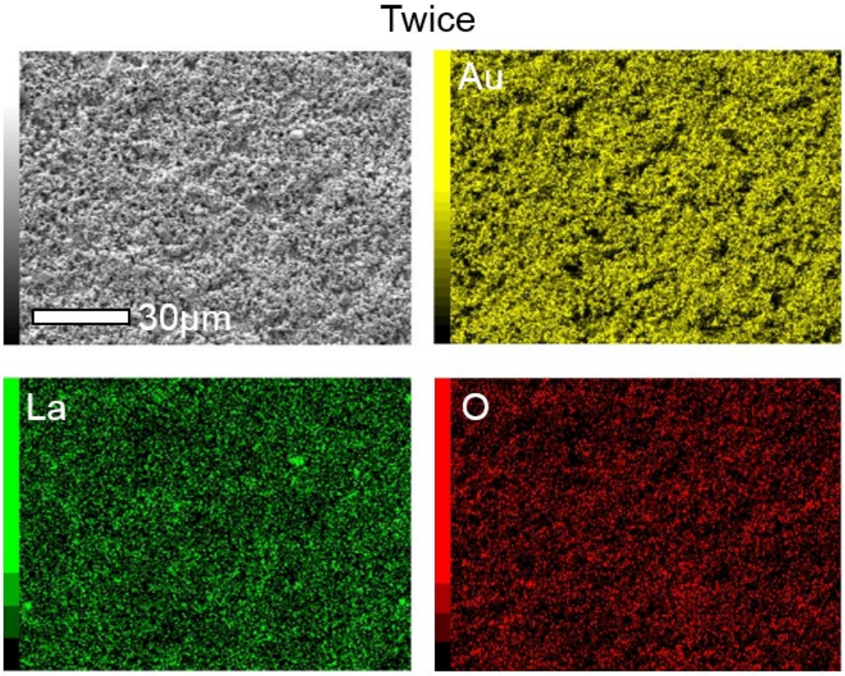

Supplementary Figure 14 Results of EDX mapping on the surface of Au electrode deposited on $LaH_{3-2x}O_x$ with $x_{nom.}$ = 0.25. The images were taken after the DC polarization measurements.

Supplementary Note 7

The apparatus for the AC impedance spectroscopy and DC polarization measurement consists of stainless steel, and the electrodes are mechanically attached to the Pd or Au electrode deposited on the sample. The SUS electrodes and the deposited electrodes are fixed by SUS screws via Al_2O_3 plates as shown below. In order to improve the contact between the SUS electrodes and deposited electrodes, Hastelloy foil is inserted between them.

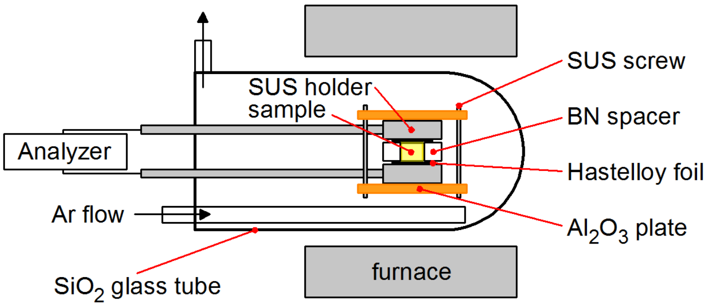

Supplementary Figure 15 Schematic of apparatus for conductivity measurement

Supplementary References

1. Bevan, D. J. M., Mohyla, J., Hoskins, B. F. & Steen, R. J. The crystal structures of some Vernier phases in the yttrium oxide-fluoride system. *Eur. J. Solid State Inorg. Chem.* **27,** 451–465 (1990).
2. Zachariasen, W. H. Crystal chemical studies of the 5f - series of elements. XIV. Oxyfluorides, XOF. *Acta Crystallogr.* **4,** 231–236 (1951).
3. Willis, B. T. M. Lattice vibrations and the accurate determination of structure factors for the elastic scattering of X-rays and neutrons. *Acta Crystallogr. Sect. A* **25,** 277–300 (1969).
4. Ashcroft, N. W. & Mermin, N. D. *Solid State Physics*. (Holt, Rinehart and Winston, 1976).
5. Lee, C. H. & Gan, C. K. Anharmonic interatomic force constants and thermal conductivity from Grüneisen parameters: An application to graphene. *Phys. Rev. B* **96,** 035105 (2017).
6. Berman, R. *Thermal conduction in solids*. (Clarendon Press, 1976).
7. Udovic, T. J., Huang, Q., Karmonik, C., Rusha, J. J. Structural ordering and dynamics of LaH_{3-x} *Journal of Alloys and Compounds* **293–295,** 113–117 (1999).
8. Harding, J. H., The calculation of free energies of point defects in ionic crystals *Physica* **131B,** 13–26 (1985).
9. Boroch, E., Conder, K., Ru-Xiu, C., Kaldis, E. An X-Ray investigation of the phase relationships in the system LaH_2−LaH_3 *J. Less Common Met.* **156,** 259–271 (1989).
10. Stecura, S. & Campbell, W. J. Thermal Expansion and Phase Inversion of Rare Earth Oxides *Bureau of Mines Report No. 5847* (United States Department of the Interior, Washington, 1961).

11. Gürel, T. & Eryiğit, R. Volume dependent vibrational properties of cerium hydrides from first principles. *J. Alloys Compd.* **477,** 478–483 (2009).
